# Pseudoprogression prediction in high grade primary CNS tumors by use of radiomics

**DOI:** 10.1038/s41598-022-09945-9

**Published:** 2022-04-08

**Authors:** Asena Petek Ari, Burak Han Akkurt, Manfred Musigmann, Orkhan Mammadov, David A. Blömer, Dilek N. G. Kasap, Dylan J. H. A. Henssen, Nabila Gala Nacul, Elisabeth Sartoretti, Thomas Sartoretti, Philipp Backhaus, Christian Thomas, Walter Stummer, Walter Heindel, Manoj Mannil

**Affiliations:** 1 University Clinic for Radiology, Westfälische Wilhelms-University Muenster and University Hospital Muenster, Albert-Schweitzer-Campus 1, 48149 Muenster, Germany; 2 Department of Medical Imaging, Radboud University Medical Center, Radboud University, 6500HB Nijmegen, The Netherlands; 3 Faculty of Medicine, University of Zurich, Zurich, Switzerland; 4 From the Institute of Diagnostic and Interventional Radiology, University Hospital Zurich, University of Zurich, Zurich, Switzerland; 5 Department of Radiology and Nuclear Medicine, Maastricht University Medical Center, Maastricht University, Maastricht, The Netherlands; 6 Department of Nuclear Medicine, Westfälische Wilhelms-University Muenster and University Hospital Muenster, Albert-Schweitzer-Campus 1, 48149 Muenster, Germany; 7 European Institute for Molecular Imaging, Westfälische Wilhelms-University Muenster and University Hospital Muenster, Albert-Schweitzer-Campus 1, 48149 Muenster, Germany; 8 Institute of Neuropathology, Westfälische Wilhelms-University Muenster and University Hospital Muenster, Albert-Schweitzer-Campus 1, 48149 Muenster, Germany; 9 Department of Neurosurgery, Westfälische Wilhelms-University Muenster and University Hospital Muenster, Albert-Schweitzer-Campus 1, 48149 Muenster, Germany

**Keywords:** CNS cancer, Prognostic markers

## Abstract

Our aim is to define the capabilities of radiomics and machine learning in predicting pseudoprogression development from pre-treatment MR images in a patient cohort diagnosed with high grade gliomas. In this retrospective analysis, we analysed 131 patients with high grade gliomas. Segmentation of the contrast enhancing parts of the tumor before administration of radio-chemotherapy was semi-automatically performed using the 3D Slicer open-source software platform (version 4.10) on T1 post contrast MR images. Imaging data was split into training data, test data and an independent validation sample at random. We extracted a total of 107 radiomic features by hand-delineated regions of interest (ROI). Feature selection and model construction were performed using Generalized Boosted Regression Models (GBM). 131 patients were included, of which 64 patients had a histopathologically proven progressive disease and 67 were diagnosed with mixed or pure pseudoprogression after initial treatment. Our Radiomics approach is able to predict the occurrence of pseudoprogression with an AUC, mean sensitivity, mean specificity and mean accuracy of 91.49% [86.27%, 95.89%], 79.92% [73.08%, 87.55%], 88.61% [85.19%, 94.44%] and 84.35% [80.19%, 90.57%] in the full development group, 78.51% [75.27%, 82.46%], 66.26% [57.95%, 73.02%], 78.31% [70.48%, 84.19%] and 72.40% [68.06%, 76.85%] in the testing group and finally 72.87% [70.18%, 76.28%], 71.75% [62.29%, 75.00%], 80.00% [69.23%, 84.62%] and 76.04% [69.90%, 80.00%] in the independent validation sample, respectively. Our results indicate that radiomics is a promising tool to predict pseudo-progression, thus potentially allowing to reduce the use of biopsies and invasive histopathology.

## Introduction

Infiltrating gliomas are high grade malignant entities, according to the World Health Organization (WHO). They entail diffuse astrocytoma (IDH mutant), anaplastic astrocytoma (IDH-mutant), glioblastoma (IDH wildtype and mutant), diffuse midline glioma (H3 K27M-mutant), oligodendroglioma (IDH mutant and 1p/19q-codeleted) and anaplastic oligodendroglioma (IDH-mutant and 1p/19q co-deleted)^1^. These malignancies are characterized by an invasive growth pattern, which results in a poor prognosis. Glioblastomas with IDH-wildtype (WHO 4) are the most common primary malignant brain tumor and account for 50–60% of all intracranial gliomas.

It has one of the worst prognoses of all oncologic entities with a median survival of 13.6 months^2^. The standard therapeutic care for these malignancies involves (partial) resection, adjuvant radiotherapy and chemotherapy with temozolomide ± lomustine. Blood–brain barrier breakdown indicated by T1 contrast-enhancement is a hallmark of glioblastoma. However, the combination of radiation and chemotherapy may also lead to contrast enhancement in MRI mimicking progression of the residual tumor, and/or the appearance of new tumor lesions^3,4^. This phenomenon is called *pseudoprogression*. Clinically, it may be associated with worsened neurological deficits, however a discrepancy between minimal clinical changes and disproportionately worsened imaging findings is more common^3^. Pseudoprogression occurs most frequently during the first three months after radiation therapy, followed by re-improvement of imaging findings after further weeks to months^5^. Because of their overlapping imaging patterns, the differentiation between true progression and pseudoprogression on MR images after chemoradiation therapy is extremely challenging. However, the accurate differentiation of these two entities is essential for selection of the optimal therapeutic strategy. Therefore, improving the accuracy of non-invasive prediction of pseudoprogression would be highly beneficial.

Radiomics represents a comprehensive quantification of medical images. It creates mineable feature spaces that can be used to non-invasively evaluate tumor heterogeneity or the underlying histopathology^6^. Due to recent advances in machine learning, radiomics may allow for personalized therapies and an improved imaging analysis beyond the scope of a visual inspection^7^. For example, recent radiomics studies showed the non-invasive prediction of histopathological tumor features, e.g. MGMT promoter methylation status^8^ and IDH mutation status^9^.

Given the potential of radiomics and the clinical importance of diagnosing pseudoprogression in patients with diffuse gliomas, we sought to define the diagnostic capacity of radiomics and machine learning in predicting pseudoprogression in a representative patient cohort diagnosed with high grade adult-type diffuse gliomas (WHO grade 3 and 4).

## Materials and methods

### Study design

The single-center study was performed in compliance with the Declaration of Helsinki and was approved by the local ethics committee (Ärztekammer Westfalen-Lippe (ÄKWL) Münster 2021-596-f-S). Due to its retrospective nature, written informed consent was waived by the local ethics committee (Ärztekammer Westfalen-Lippe (ÄKWL) Münster 2021-596-f-S). We retrospectively screened our databases at the Department of Radiology, Nuclear medicine and Neuropathology for patients with histologically-proven high-grade gliomas, who were presented to our tertiary referral hospital between January 2015 and June 2020.

From the initially detected 193 patients we excluded those with (1) missing or non-diagnostic pre-treatment cerebral magnetic resonance imaging (n = 26), (2) insufficient diagnostic imaging quality (n = 2), (3) inconsistent histopathology (n = 3) and (4) insufficient follow-up examinations (n = 31).

Finally, 131 patients were included, of which 64 patients had a histopathologically proven progressive disease (PD) and 67 were diagnosed with mixed or pure pseudoprogression (PsP) after initial treatment.

Clinical and imaging data of each individual patient was reviewed for histopathological subtypes such as IDH-, MGMT-methylation and ATRX-Status and used therapy scheme.

### Image data

Multivendor T1-weighted post contrast images of the included patients were obtained at different centers and magnetic field strengths (either 1.5 T or 3.0 T).

The images were available for assessment via our local picture archiving and communication system. The studies were evaluated for completeness and image quality by two experienced neuroradiologists (nine and two years of experience).

### Radiomics

From the available pre-treatment diagnostic magnetic resonance images, we collected the entire image stack of the contrast-enhanced T1-weighted images (CE-T1WI) in Digital Imaging and Communications in Medicine (DICOM) format.

Segmentation of the enhancing parts of the tumor was semi-automatically performed by the above mentioned experienced neuroradiologists using the 3D Slicer open-source software platform (version 4.10, www.slicer.org) and utilizing the Segmentation Wizard plugin. Consensus was achieved in cases of differing extent of segmentation.

We performed a standardized preprocessing step on all images: first spatial resampling to 2 × 2 × 2 voxels, then a bin width of 64 was set.

For the computation of the radiomics features we used the open source PyRadiomics package available as an implementable plugin into the 3D Slicer platform.

Finally, 107 radiomic features were calculated for seven different features classes: 18 first order statistics, 14 shape-based features, 24 Gy level co-occurrence matrix, 16 Gy level run length matrix, 16 Gy level size zone matrix, 5 neighboring gray tone difference matrix and 14 Gy level dependence matrix features.

### Statistical analysis

Statistical analysis was performed using R software (version 3.5.3). We allocated the 131 patients to training data, test data and an independent validation sample at random. We denoted the training data together with the test data as the development sample. The development sample was used to construct the models and to optimize the tuning parameters included in the models. The performance of the models was determined with the validation sample (i.e. using unknown/ independent data). A stratified 4:1 ratio (development sample: 106 patients, validation sample: 25 patients) was used with the distribution of tumor progress (yes/ no) and gender (female/ male) kept balanced between both samples (Table 1). All Radiomics features underwent a Yeo-Johnson transformation in order to make the data more normal distribution-like. They were z-score normalized and then subjected to a 95% correlation filter keeping 54 features to account for redundancy between the features. The feature selection and model construction were performed with the development sample, using Generalized Boosted Regression Models (GBM). A GBM is a combination of a decision tree algorithm and a boosting technique. Usually, GBM prediction models are constructed as an ensemble of weak predictions models (weak learners).

**Table 1:** Histopathological diagnosis and demographic data.

	Development data	Validation data
Number	106	25
**Progress**		
Yes (%)	49.06	48.00
No (%)	50.94	52.00
**Gender (%)**		
Male	56,60	56,00
Female	43.40	44.00
Age (years)	61.18	59.04

First, we performed a GBM to identify the 15 most important features. These 15 most important variables are listed in Table 2. We created our model with an increasing number of these previously identified features. Initially, the model contained only the most important feature (“orig.ngtdm.Strength”). Subsequently, we added one feature at a time. The model with the highest performance with respect to the test data set is used as the final model. This step-by-step approach determined the final number of features included in the model.

**Table 2:** Feature selection: most important Radiomics features (in descending order of importance).

Level of importance	Feature
1	orig.ngtdm.Strength
2	Age
3	orig.glcm.ClusterShade
4	orig.shape.MinorAxisLength
5	orig.shape.Elongation
6	orig.glrlm.LongRunHighGrayLevelEmphasis
7	orig.ngtdm.Busyness
8	orig.shape.Sphericity
9	orig.glcm.Imc2
10	orig.glszm.LowGrayLevelZoneEmphasis
11	orig.glcm.MCC
12	orig.fst.ord.RobustMeanAbsoluteDeviation
13	orig.fst.ord.Median
14	orig.gldm.LowGrayLevelEmphasis
15	orig.ngtdm.Contrast

The GBM models contain several tuning parameters: firstly the “tree depth”, secondly the “learning rate”, thirdly the “minimum number of observations in the terminal node” and finally the “number of trees”. These tuning parameters of the GBM models (tree depth = 1 or 2; learning rate = 0.01 or 0.1; minimum number of observations in terminal nodes = 5,7,9,11,13 or 15, number of trees = 125) were determined using a tenfold cross validation (i.e. we divided the development sample 10 times into 90% training data and 10% unseen test data). This technique ensures that the training and test sample do not overlap. This is a methodology used to obtain robust results with small datasets. To determine the stability of the results, each of the models (with a given number of features) was optimized 100 times. The predictive power of each model was analyzed using the area under the curve (AUC) of the receiver operator characteristic (ROC) and the accuracy (both as the mean of the 100 cycles/ repetitions with cross validation).

## Results

Our cohort included 131 patients (male: n = 74; female: n = 57), diagnosed with progress (n = 64) and pseudoprogress (n = 67) of the primary brain tumor. The mean age of our patient cohort was 60.77 years. The histopathological diagnosis and demographic data of the development group and the validation group are summarized in Table 1. A GBM model was used for the feature selection and for the subsequent model construction. Starting with the most important of the original 54 features (i.e. the feature “orig.ngtdm.Strength”), we added one additional feature in each subsequent step.

**Figure 1:**
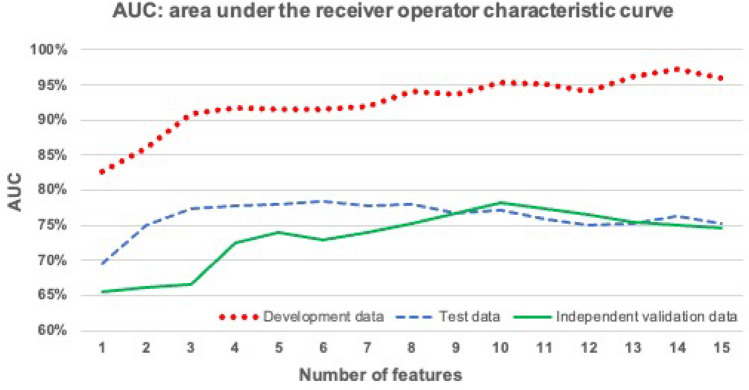
Mean area under the curve (AUCs) of 100 cycles for the GBM models with ascending number of Radiomics features.

**Figure 2:**
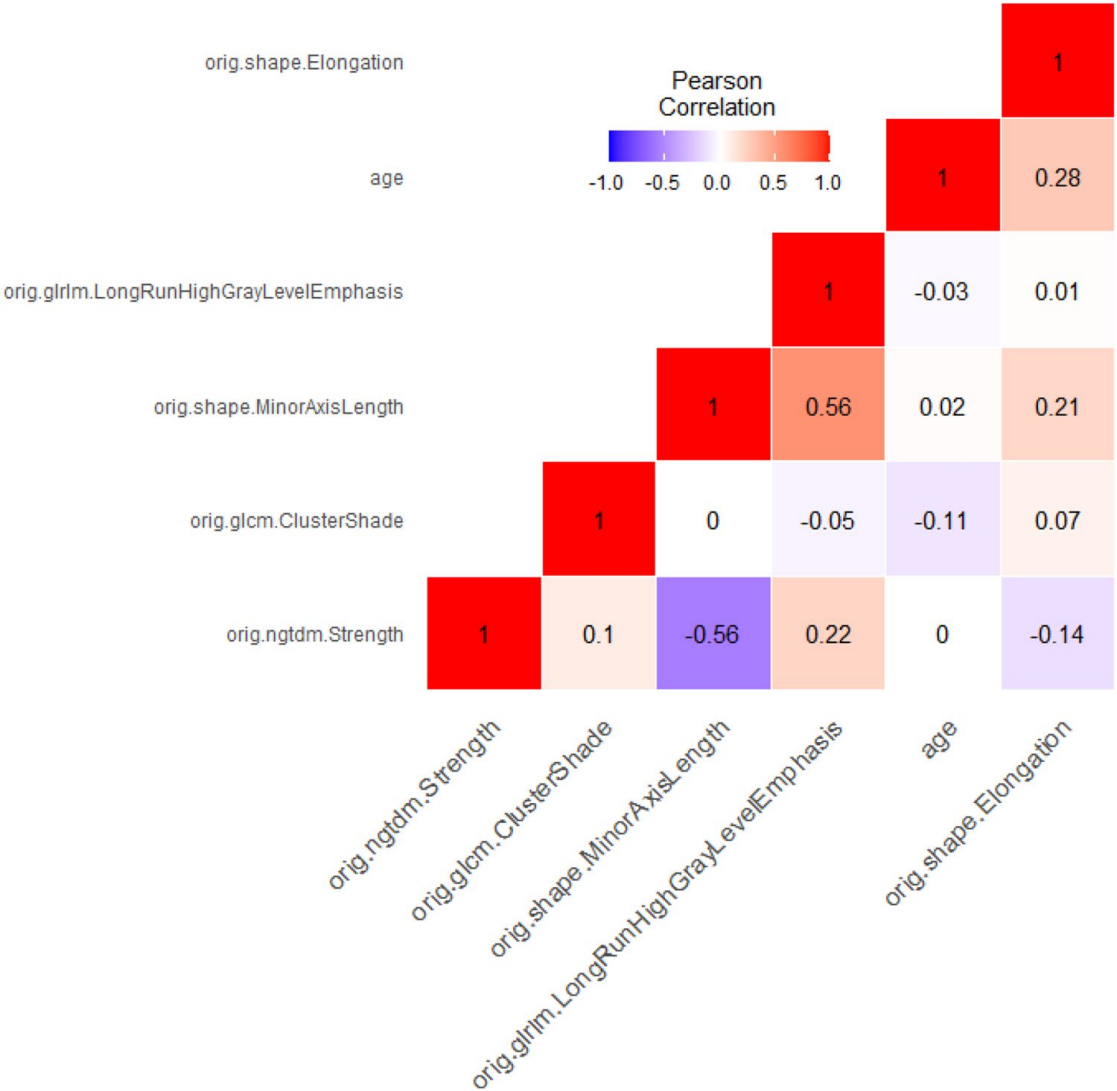
Pearson Correlation for selected GBM model with 6 features.

**Figure 3:**
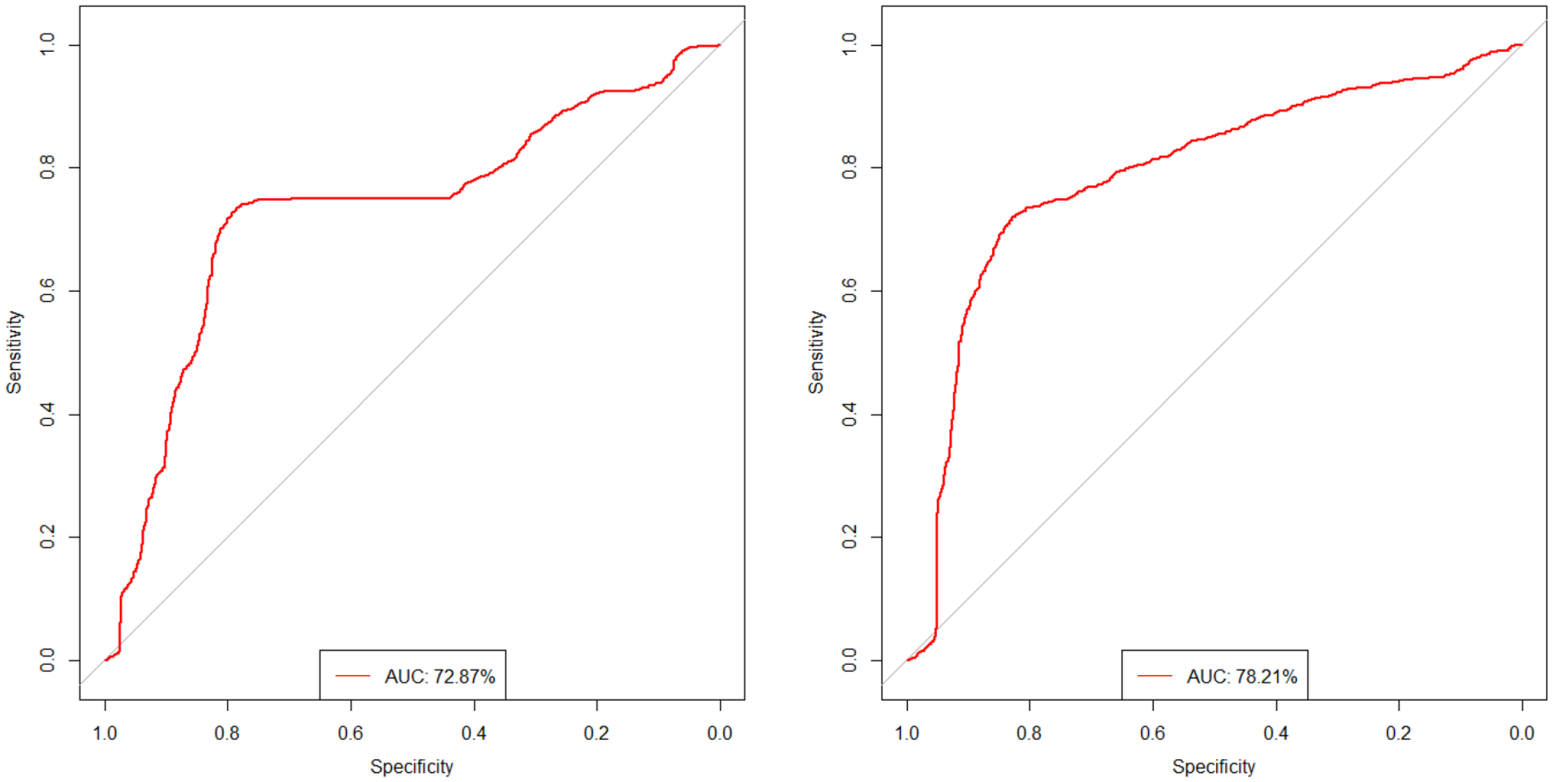
ROC curves of the validation group for GBM model with six features (left) and ten features (right).

The optimization of each of these GBM models was repeated 100 times using tenfold cross-validation. The results (for each model averaged over 100 cycles) are summarized in Table 3. The performance of the models depended only to a limited extent on the number of features used. It is interesting to observe that similar performances are obtained with the unseen test sample and the independent validation sample. The best models in terms of AUC were obtained with six features (Fig. 1). The correlation matrix for the best model (including the last six features) is shown in Fig. 2. The small absolute values of most of the correlation coefficients indicate that the features used in this model were majorly independent of each other. The mean AUC, sensitivity, specificity and accuracy of this model for predicting true progression in the testing group were 78.51% [75.27%, 82.46%], 66.26% [57.95%, 73.02%], 78.31% [70.48%, 84.19%] and 72.40% [68.06%, 76.85%], respectively (brackets indicate the 95% confidence intervals). In the independent validation group, the mean AUC, sensitivity, specificity and accuracy were 72.87% [70.18%, 76.28%], 71.75% [62.29%, 75.00%], 80.00% [69.23%, 84.62%] and 76.04% [69.90%, 80.00%] and finally in the full development group 91.49% [86.27%, 95.89%], 79.92% [73.08%, 87.55%], 88.61% [85.19%, 94.44%] and 84.35% [80.19%, 90.57%], respectively. Hence, this final GBM model showed similar good prediction performance in the test and validation group. The model with ten features achieved a slightly higher discriminatory power on the validation data. The mean AUC, mean sensitivity, mean specificity and mean accuracy of this model were 78.21% [73.72%, 82.39%], 71.67% [58.33%, 83.33%], 82.85% [69.23%, 92.31%] and 77.48% [69.90%, 84.00%]. Figure 3 shows the receiver operating characteristic (ROC) curves of the two models with six and ten features for the independent validation group.

**Table 3:** Classification results per group.

Number of features	Test data				Development data				Independent validation data			
	AUC (%)	Sens. (%)	Spec. (%)	Acc. (%)	AUC (%)	Sens. (%)	Spec. (%)	Acc. (%)	AUC (%)	Sens. (%)	Spec. (%)	Acc. (%)
1	69.50	52.94	75.29	64.32	82.55	61.58	88.70	75.40	65.46	62.50	67.15	64.92
2	74.98	63.18	77.72	70.59	86.06	69.38	83.22	76.43	66.16	53.58	73.00	63.68
3	77.26	65.65	76.10	70.97	91.00	79.04	87.61	83.41	66.59	68.33	72.92	70.72
4	77.79	66.02	75.37	70.78	91.74	81.81	89.13	85.54	72.51	70.00	78.46	74.40
5	78.04	66.39	77.21	71.90	91.50	80.25	88.98	84.70	73.91	74.17	75.38	74.80
6	**78.51**	66.26	78.31	**72.40**	91.49	79.92	88.61	84.35	72.87	71.75	80.00	76.04
7	77.75	65.90	77.89	72.01	91.90	80.98	89.65	85.40	73.89	72.75	82.23	77.68
8	78.06	68.21	76.04	72.20	94.02	83.65	91.93	87.87	75.28	73.42	82.31	78.04
9	76.63	66.47	75.22	70.92	93.71	83.17	91.78	87.56	76.72	71.75	80.85	76.48
10	77.09	67.44	74.38	70.98	95.44	85.83	92.96	89.46	**78.21**	71.67	82.85	77.48
11	75.88	66.36	72.95	69.72	95.21	86.35	93.02	89.75	77.42	71.08	81.54	76.52
12	75.12	65.31	72.80	69.13	94.04	84.04	91.37	87.77	76.49	69.83	80.38	75.32
13	75.26	65.69	71.94	68.87	96.09	88.02	94.06	91.09	75.37	69.58	82.15	76.12
14	76.28	66.39	72.09	69.30	97.19	90.63	95.69	93.21	75.13	69.00	82.85	76.20
15	75.29	64.89	72.09	68.56	96.03	89.13	94.50	91.87	74.60	68.00	80.54	74.52

*AUC* area under the curve (receiver operator characteristics, *Sens.* Sensitivity, *Spec.* specificity, *Acc.* accuracy.

Significance values are in bold.

## Discussion

Our radiomics approach with only six features was able to predict the occurrence of pseudoprogression with an AUC, mean sensitivity, mean specificity and mean accuracy of 91.49% [86.27%, 95.89%], 79.92% [73.08%, 87.55%], 88.61% [85.19%, 94.44%] and 84.35% [80.19%, 90.57%] in the full development group and 72.87% [70.18%, 76.28%], 71.75% [62.29%, 75.00%], 80.00% [69.23%, 84.62%] and 76.04% [69.90%, 80.00%] in the independent validation group, respectively.

The detection of pseudoprogression after radiation therapy is an important clinical problem. Conventional MRI including pre- and post-contrast T1 weighted images remains the most common diagnostic method^10^, limitations persist in enabling an accurate and reliable differentiation of true progression from pseudoprogression^11^. Recent studies have confirmed the added value of advanced imaging methods, including spectroscopy, amino acid PET and perfusion MRI, to improve the differentiation of these two entities^12–15^. However, availability, scan time restrictions, reimbursement issues and a lack of standardization limit the widespread clinical use of such advanced imaging methods.

In clinical routine physicians often resort to a combination of imaging and biopsy to ascertain the final diagnosis of true progression or pseudoprogression, as this combination is considered the gold standard with the highest diagnostic accuracy^16^. However, the invasive nature of biopsy harbors inherent risks for complications.

Several studies have shown the potential of radiomics for adding important diagnostic information to HGG diagnosis and prognosis. For instance, based on combining selected MRI radiomics, genetic and clinical risk factors, Tan et al. predicted the overall survival using contrast enhanced T1 weighted and T2/ FLAIR weighted MR images^17^. Zhang et al. predicted the IDH genotype in high-grade gliomas with an accuracy of 89% in the validation dataset^9^. Similarly, Zhou et al. extracted features from conventional MR images of more than 500 patients with diffuse low- and high-grade gliomas and predicted IDH mutation and 1p19q codeletion status^18^. Chiu et al. designed a radiomic-based model with MRIs for the efficient classification of tumor subregions of GBM^19^. Based on several MRIs, Tian et al. evaluate TERT (telomerase reverse transcriptase) promoter mutations in HGG by using radiomics and detected relevant indicators (Age, Cho/Cr, Lac, CNV, and Radscore)^20^.

However, to the best of our knowledge, no other study used this technique to predict the occurrence of pseudoprogression with a similar sample size or similar methodology.

Most importantly, we would like to highlight that in this study special consideration was given towards minimizing overfitting in the ML-backed prediction model. Specifically, we divided the data into a development sample, which was trained 10 times into 90% training data and 10% unseen test data and repeated 100 cycles to determine the mean score each time. We then validated our results in another previously unseen data set. Interestingly by using GBM, we get similar results with the unseen test sample and with the truly independent validation sample. This further corroborates the reliability and reproducibility of our results.

This study has several limitations that need to be addressed. Firstly, this was a retrospective study with inherent limitations. Secondly, we did not include diffuse astrocytic and oligodendroglial CNS tumors or include equal number of patients with different mutations. Furthermore, we had to excluded 62 patients due to various reasons. Lastly, our independent, previously unseen validation data set was relatively small. Larger prospective cohorts are required to confirm our findings.

Despite these limitations, we obtained robust results with a relatively small dataset using an independent external validation data set.

In conclusion, our results indicate that radiomics is a promising tool to predict the occurrence of pseudoprogression, thus potentially allowing physicians to reduce the use of biopsies and invasive histopathology. However, further prospective clinical data are needed before this technique can be translated into clinical practice.
